# Assessing crystal field and magnetic interactions in diuranium-μ-chalcogenide triamidoamine complexes with U^IV^–E–U^IV^ cores (E = S, Se, Te): implications for determining the presence or absence of actinide–actinide magnetic exchange†
†Electronic supplementary information (ESI) available: Supplementary magnetic data and modelling, and crystallographic details. CCDC 1547173–1547177. For ESI and crystallographic data in CIF or other electronic format see DOI: 10.1039/c7sc01998j



**DOI:** 10.1039/c7sc01998j

**Published:** 2017-07-05

**Authors:** Benedict M. Gardner, David M. King, Floriana Tuna, Ashley J. Wooles, Nicholas F. Chilton, Stephen T. Liddle

**Affiliations:** a School of Chemistry , The University of Manchester , Oxford Road , Manchester , M13 9PL , UK . Email: steve.liddle@manchester.ac.uk ; Email: nicholas.chilton@manchester.ac.uk; b School of Chemistry , The University of Nottingham , University Park , Nottingham , NG7 2RD , UK

## Abstract

We report the synthesis and characterisation of a family of diuranium(iv)-μ-chalcogenide complexes including a detailed examination of their electronic structures and magnetic behaviours. Treatment of [U(Tren^TIPS^)] [**1**, Tren^TIPS^ = N(CH_2_CH_2_NSiPr^i^_3_)_3_] with Ph_3_PS, selenium or tellurium affords the diuranium(iv)-sulfide, selenide, and telluride complexes [{U(Tren^TIPS^)}_2_(μ-E)] (E = S, **2**; Se, **5**; Te, **6**). Complex **2** is also formed by treatment of [U(Tren^TIPS^){OP(NMe_2_)_3_}] (**3**) with Ph_3_PS, whereas treatment of **3** with elemental sulfur gives the diuranium(iv)-persulfido complex [{U(Tren^TIPS^)}_2_(μ-η^2^:η^2^-S_2_)] (**4**). Complexes **2–6** have been variously characterised by single crystal X-ray diffraction, NMR, IR, and optical spectroscopies, room temperature Evans and variable temperature SQUID magnetometry, elemental analyses, and complete active space self consistent field spin orbit calculations. The combined characterisation data present a self-consistent picture of the electronic structure and magnetism of **2**, **5**, and **6**, leading to the conclusion that single-ion crystal field effects, and not diuranium magnetic coupling, are responsible for features in their variable-temperature magnetisation data. The presence of magnetic coupling is often implied and sometimes quantified by such data, and so this study highlights the importance of evaluating other factors, such as crystal field effects, that can produce similar magnetic observables, and to thus avoid misassignments of such phenomena.

## Abstract 2

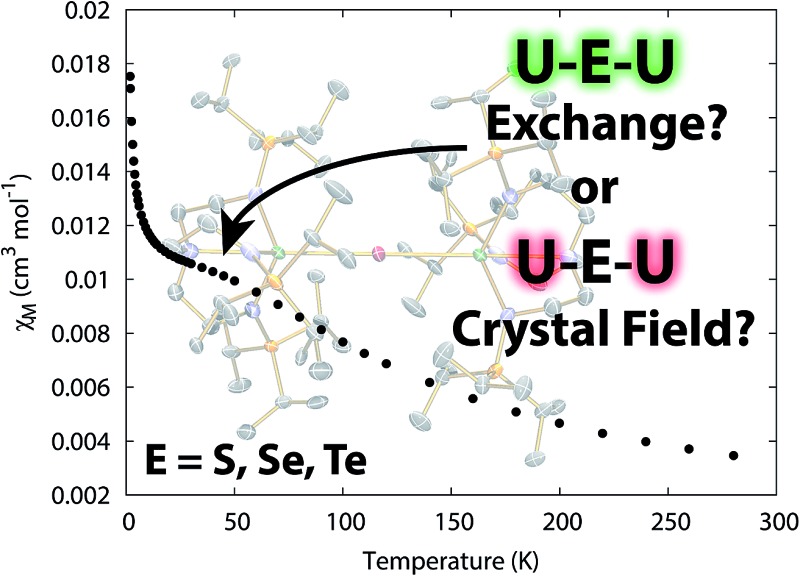
Analysis of U^IV^–E–U^IV^ (E = S, Se, Te) complexes reveals their behaviour is due to crystal field effects and not exchange coupling.

## Introduction

There is continued interest in understanding the magnetic behaviour of actinide compounds,^1^ in particular multimetallic complexes that may exhibit magnetic exchange interactions, in order to gain detailed knowledge of their electronic structure that underpins their unique chemical reactivity.^2^ However, unravelling their behaviour is complicated considerably by the rich interplay of interelectronic repulsion (IER), spin orbit coupling (SOC), and crystal field (CF) effects.^3^ For uranium in particular, the strength of each of these components of electronic structure can vary significantly depending on the oxidation state of uranium and nature of the coordinated ligands,^4^ making comprehensive analysis a significant challenge even with modern characterisation and computational techniques. Where magnetic exchange interactions involving uranium are concerned, detecting their presence by variable temperature magnetometry is difficult because of the non-trivial temperature dependence arising from the CF and SOC states of the individual magnetic centres obscuring possible magnetic exchange. Spectroscopic methods (*i.e.* electron paramagnetic resonance, EPR) for determination of exchange is much more reliable,^5^ however this is not amenable in all cases such as when the exchange is relatively strong (*J* ≫ *hν*) or for non-Kramers ions like uranium(iv) where singlet states usually give EPR-silent species.^6^

Although clear-cut examples of magnetic exchange in uranium complexes remain rare, the majority of documented cases involve uranium(v),^7–12^ and there are few reports of magnetic exchange in diuranium(iv) complexes or uranium(iv)–transition metal coupling.^13–19^ Where magnetic exchange for actinide complexes is proposed this is usually on the basis of observing a maxima in the magnetic susceptibility (*χ*) *vs. T* plot (often referred to as the Néel point),^20,21^ however care needs to be taken when using this as evidence alone. If a clear maximum is observed this is usually due to antiferromagnetic exchange, however if the maximum is obscured in any way,^22–26^ for example by a low-level (even ∼1%) paramagnetic impurity which becomes prominent at low temperature, then the presence or not of magnetic coupling becomes nebulous and cannot be stated with confidence;^27^ however, alternative explanations are often not immediately obvious. An alternative and rarely invoked explanation for ambiguous plateaued maxima/shoulders in *χ vs. T* plots could in fact, rather than owing to magnetic exchange, simply reflect single-ion CF effects; this has rarely been investigated in detail since families of isostructural diuranium molecules with a systematic variation of bridging groups are few in number.

Recently we have reported a range of uranium-group 15 element bonds varying from formal single to triple bond interactions.^28–34^ These complexes have been supported by the sterically demanding ligand N(CH_2_CH_2_NSiPr^i^_3_)_3_ (Tren^TIPS^), which also supported the synthesis of a terminal mono(oxo) complex.^35^ Seeking to extend our range of chalcogenide complexes we extended our studies to heavier chalcogens, noting that from related group 15 and thorium derivatives,^28–34,36–39^ and recent reports of diuranium-chalcogenide complexes,^14,40–44^ that complexes with bridging U–E–U cores were likely to result (E = S, Se, Te). We reasoned that this would present a family of U–E–U complexes with which to systematically probe their electronic structure and magnetism.

Here, we report on the synthesis and magnetism of U–E–U complexes supported by Tren^TIPS^, and show that shoulders that could be interpreted as local maxima in magnetometry data, which could be attributed to magnetic exchange, are in fact almost certainly the result of single-ion CF effects. This gives a greater appreciation of the CF effects that are intimately involved in unravelling the magnetic properties, and therefore electronic structure, of actinide complexes, and specifically for the identification of magnetic exchange effects; this is of pre-eminent importance to progressing the field of actinide molecular magnetism and improving our understanding of actinide electronic structure and correlating this to chemical reactivity.

## Results and discussion

### Synthesis and characterisation of complexes **2–6**

**Scheme 1:**
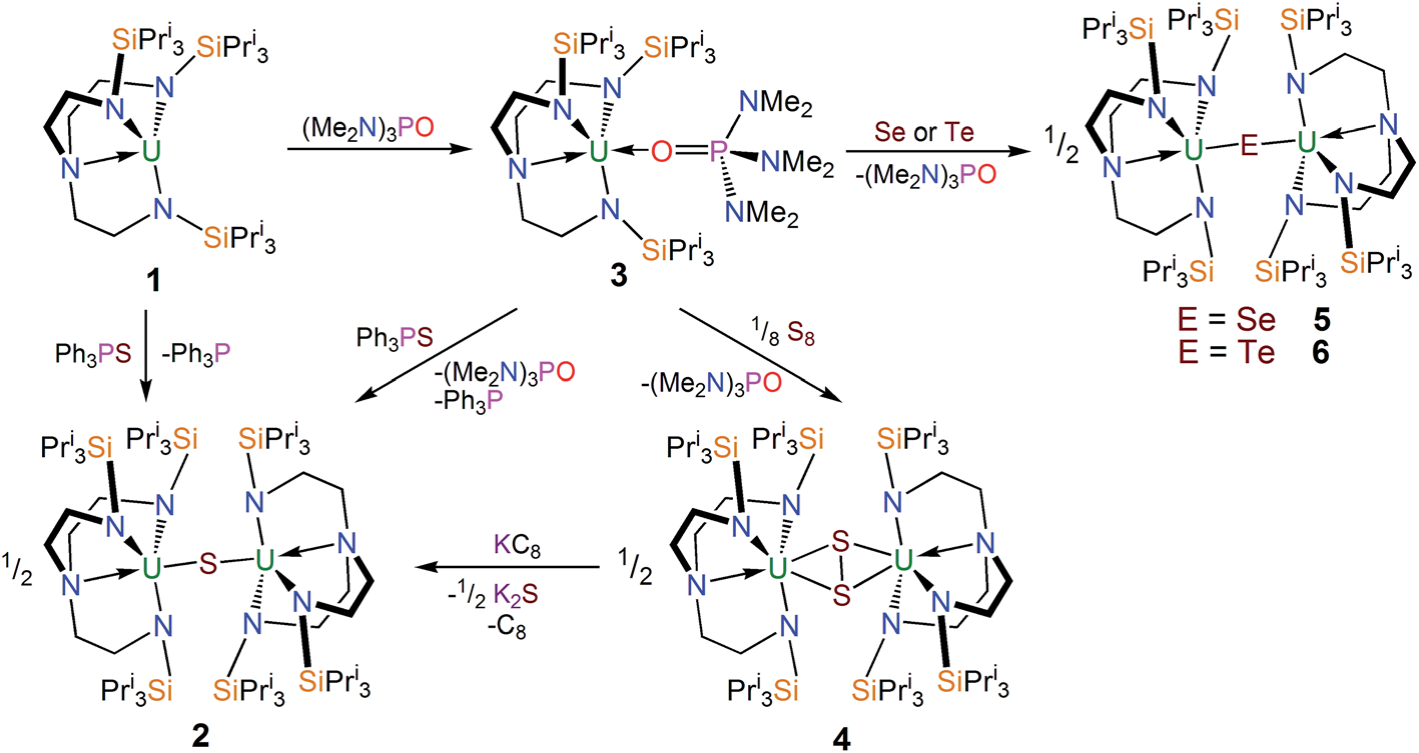
Synthesis of **2–6**.

**Fig. 1:**
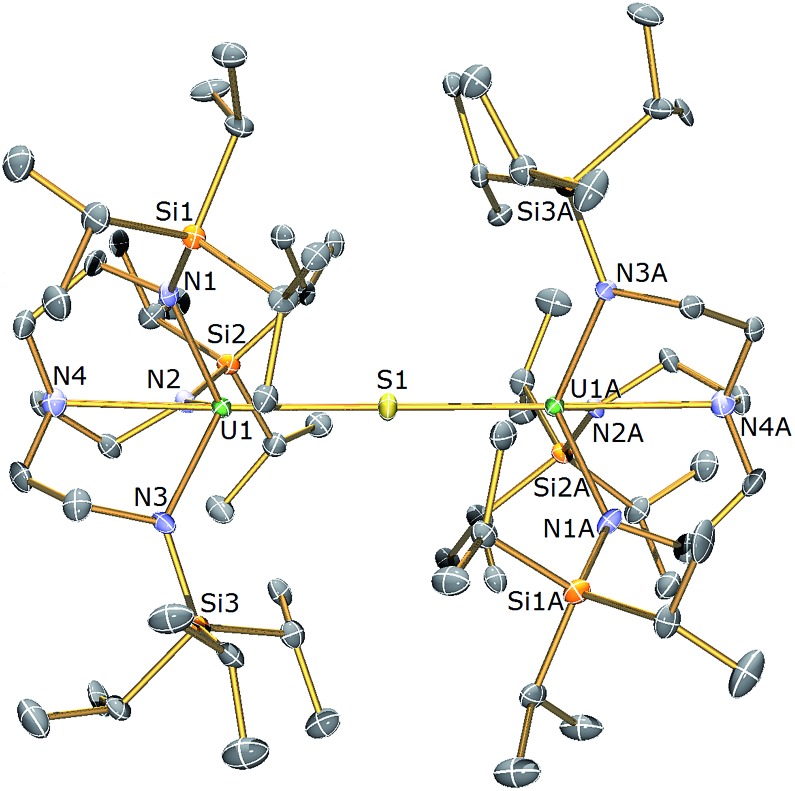
Molecular structure of **2**. Displacement ellipsoids set at 40% probability. Lattice THF solvent molecules and H atoms are omitted for clarity. Selected bond distances (Å) and angles (°): U1–S1 2.6903(6), U1–N1 2.253(8), U1–N2 2.259(7), U1–N3 2.261(8), U1–N4 2.704(7), U1–S1–U1A 179.81(18), S1–U1–N4 178.18(19).

The uranium(iii) complex [U(Tren^TIPS^)]^28^ [**1**, Tren^TIPS^ = N(CH_2_CH_2_NSiPr^i^_3_)_3_] reacts with Ph_3_PS (Scheme 1) in toluene to afford a yellow precipitate. The remaining solution contains significant quantities of unreacted Ph_3_PS from a 1 : 1 reaction – as ascertained by removal of solvent *in vacuo* and analysing the residue by ^1^H NMR spectroscopy – but no resonances attributable to **1** were observed. The ^1^H NMR spectrum of the yellow precipitate suggested the formation of a new complex and crystallisation from THF afforded crystals of sufficient quality for a single crystal X-ray diffraction (XRD) study, which confirmed the formulation to be [{U(Tren^TIPS^)}_2_(μ-S)] (**2**). The molecular structure of **2** is illustrated in Fig. 1 with selected bond lengths and angles.

The solid state molecular structure of **2** reveals a dinuclear Tren^TIPS^–uranium μ-sulfide complex with the S-centre bridging the two uranium ions. The five-coordinate uranium centres are each coordinated by one tetradentate Tren^TIPS^ ligand and the bridging sulfide, the latter bridging the two U atoms with a linear U–S–U bond angle [179.81(18)°]. As a result of the sterically demanding Tren^TIPS^ ligands the uranium centres adopt distorted trigonal bipyramidal geometries and exhibit U–N bond distances that are typical of uranium(iv)–N_amide_ and –N_amine_ bonds.^45^ The two Tren^TIPS^ ligands in **2** adopt an eclipsed orientation when viewed along the U–S–U bond vector. The two identical U–S bond distances in **2** of 2.6903(6) Å lie at the upper end of the range of bridging U^IV^–S bonds in reported examples [2.588(1)–2.713(2) Å],^40–44^ alluding to the sterically encumbered nature of Tren^TIPS^, but are slightly shorter than the sum of the covalent single bond radii of uranium and sulfur (2.73 Å),^46^ supporting the presence of a U^IV^–S–U^IV^ unit.

The ^1^H NMR spectrum of **2** features seven paramagnetically shifted resonances in the range –22 to +7 ppm, presumably as a consequence of low symmetry from the eclipsed orientation of the Tren^TIPS^ ligands observed in the solid state being maintained in solution on the NMR timescale. The room temperature solution magnetic moment (Evans method) of **2** in benzene of 3.79 *μ*_B_ is consistent with the presence of two 5f^2^ uranium(iv) centres.^47^ Variable temperature magnetometry carried out on a solid sample of **2** revealed a *μ*_eff_ value of 3.47 *μ*_B_ at 300 K that is in good agreement with the solution magnetic moment considering the difference of phases. This value decreases steadily to 3.25 *μ*_B_ at 100 K at which point the value of *μ*_eff_ drops more sharply to 0.61 *μ*_B_ at 1.8 K (Fig. 8). It is worth highlighting at this juncture that the uranium(iv) ions in **2** clearly adopt magnetic singlet ground states with modest temperature independent paramagnetism contributions at low temperature which, from a simple CF-only approximation, would be anticipated for an O_h_ ligand field. The trigonal bipyramidal ligand field of these complexes predicts a magnetically active E ground state,^13^ but all Tren–uranium(iv) complexes exhibit magnetisation behaviours consistent with A ground states; this suggests that SOC effects dominate over the CF, which is borne out by our modelling (see below).

**Fig. 2:**
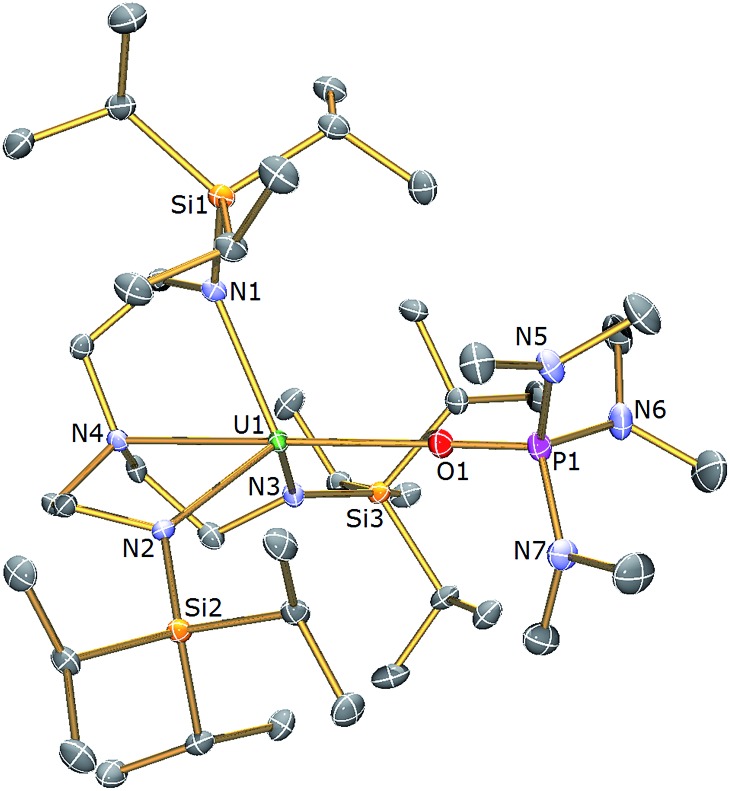
Molecular structure of **3**. Displacement ellipsoids set at 40% probability. H atoms are omitted for clarity. Selected bond distances (Å) and angles (°): U1–O1 2.498(3), U1–N1 2.390(4), U1–N2 2.369(4), U1–N3 2.375(4), U1–N4 2.699(4), P1–O1 1.504(3), N4–U1–O1 174.73(11), U1–O1–P1 176.3(2).

The isolation of a diuranium(iv)-sulfide suggests that despite the appropriate reagent stoichiometry for the anticipated two-electron oxidation reaction, *i.e.* 1 + 1/8 S_8_ to form “[(Tren^TIPS^)U^V^ =(S)]”, kinetic trapping of the postulated terminal sulfide complex by available **1** in solution with disproportionation of U^V^ =S···U^III^ to U^IV^–S–U^IV^ ought to be considered likely. It should be noted that Tren^TIPS^ has proven capable of stabilising terminal uranium(v/vi)-nitrides and parent uranium(iv)-imide, -phosphinidene, and -arsinidene complexes,^5,28–35^ but although these complexes suggest that stabilisation of a terminal sulfide should be possible^48–52^ with Tren^TIPS^, the nitride is small compared to sulfide, whereas the pnictidenes are anionic formulations that would resist the formation of bridging species. Further, although dithorium bridging pnictido and pnictidiide complexes supported by Tren^TIPS^ have been reported recently,^36,37^ the analogous uranium complexes are yet to be reported which may be related to the larger size of thorium compared to uranium. In an attempt to counteract μ-sulfide formation, employment of a Lewis base adduct of **1** was envisaged to limit the quantity of free **1** available and thus permit isolation of a terminal sulfide complex. Thus **1** was treated with one equivalent of (Me_2_N)_3_PO (HMPA, Scheme 1), to afford a dark green precipitate whose ^1^H NMR spectrum indicated the formation of a new complex with threefold molecular symmetry in solution on the NMR timescale at room temperature. Crystallisation from hexanes afforded crystals of suitable quality for a single crystal XRD study, which confirmed the formulation to be [U(Tren^TIPS^)(HMPA)] (**3**). The molecular structure of **3** is illustrated in Fig. 2 with selected bond lengths and angles.

The uranium centre in the solid state structure of **3** adopts a distorted trigonal bipyramidal geometry and is coordinated to the tetradentate Tren^TIPS^ ligand and the oxygen atom of an HMPA ligand. The U–N_amide_ and U–N_amine_ bond distances in **3** of 2.378(4) (av.) and 2.699(4) Å lie are as expected extended by ∼0.05 and ∼0.14 Å, respectively, relative to HMPA-free **1**.^28^ The U1–O1 bond distance of 2.498(3) Å is comparable to that observed in [U(Tren^DMBS^)(HMPA)] [Tren^DMBS^ = N(CH_2_CH_2_NSiMe_2_Bu^*t*^)_3_] (2.460(3) Å),^53^ which features a uranium(iii) ion. The structural parameters in the remainder of the HMPA ligand are unexceptional and the N_amine_–U–O angle is essentially linear [N4–U1–O1: 174.73(11) Å].

The ^1^H NMR spectrum of **3** features four paramagnetically shifted resonances in the range –4 to +6 ppm, reflecting the *C*_3_ symmetry, and one additional resonance at 1.10 ppm assigned to the methyl groups present in the coordinated HMPA. The ^31^P{^1^H} NMR spectrum reveals a single phosphorus environment at +90.0 ppm which is significantly shifted downfield from that of uncoordinated HMPA (*δ* 24.6 ppm) and suggests polarisation of electron density away from the P centre towards the uranium centre in **3**, consistent with a [(Me_2_N)_3_PO→U(Tren^TIPS^)] donor interaction.

The room temperature solution magnetic moment (Evans method) of **3** in benzene of 3.02 *μ*_B_ compares with a value of 2.80 *μ*_B_ for the Tren–uranium(iii) complex [U(Tren^DMBS^)(HMPA)] and 2.85 *μ*_B_ for **1**.^28,53^ Variable temperature magnetometry of a solid sample of **3** (Fig. S1†) revealed a *μ*_eff_ value of 2.74 *μ*_B_ at 300 K. These data are lower than the theoretically expected moment of 3.69 *μ*_B_ for a 5f^3^ configuration with a ^4^I_9/2_ electronic ground state due to CF effects, and are consistent with reported uranium(iii) complexes.^47^ This value decreases steadily to ∼50 K at which point the value of *μ*_eff_ drops off more sharply to 1.55 *μ*_B_ at 1.8 K. This behaviour is characteristic of 5f^3^ uranium(iii), with magnetic moments of above 1 *μ*_B_ at low temperature due to the Kramers doublet ground state.

**Fig. 3:**
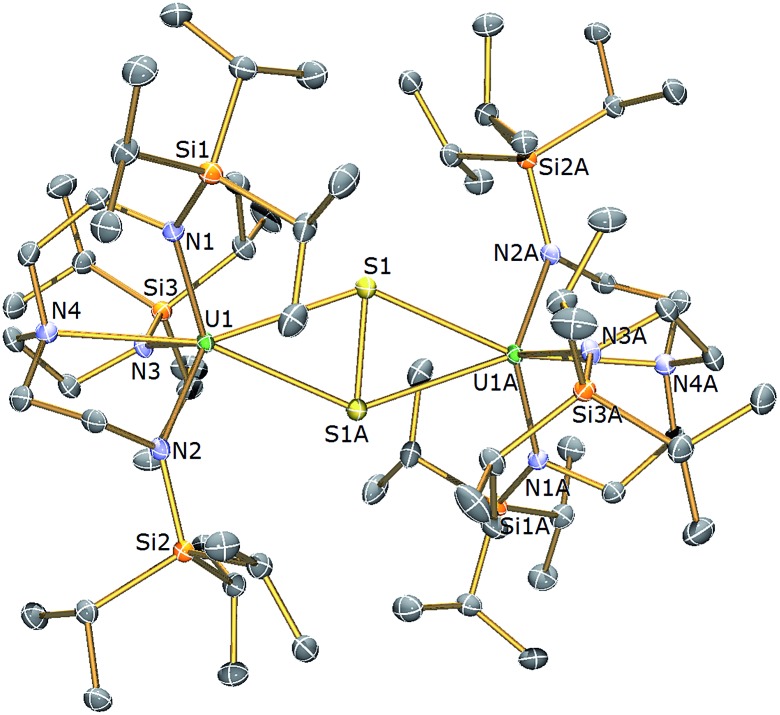
Molecular structure of **4**. Displacement ellipsoids set at 40% probability. H atoms are omitted for clarity. Selected bond distances (Å) and angles (°): U1–S1 2.8666(12), U1–S1A 2.9280(15), U1A–S1 2.9280(15), U1–S1A 2.8666(12), U1–N1 2.254(3), U1–N2 2.270(3), U1–N3 2.268(3), U1–N4 2.709(3), S1–S1A 2.1041(18), U1–S1–U1A 137.43(4), S1–U1–S1 42.57(4), S1–S1A–U1 70.27(5), S1–S1A–U1A 67.16(4).

With complex **3** in hand, a terminal uranium-sulfide species was targeted for comparative purposes by treatment of **3** with Ph_3_PS (Scheme 1). However, following work-up, **2** was isolated as the exclusive uranium product. We suggest that a terminal sulfide complex may be formed transiently in the reaction of **3** with Ph_3_PS – as additionally the production of PPh_3_ is observed – but it is a sufficiently strong Lewis base to displace HMPA from **3** and form the isolable bridging sulfide complex **2**. Reasoning that an alternative sulfur reagent may lead to differing reactivity, **3** was treated with 0.125 equivalents of S_8_ in toluene (Scheme 1), which afforded, after work up and crystallisation, orange crystals suitable for a single crystal XRD study that confirmed the structure to be [{U(Tren^TIPS^)}_2_(μ-η^2^:η^2^-S_2_)] (**4**). The molecular structure of **4** is illustrated in Fig. 3 with selected bond lengths and angles.

The molecular structure of **4** reveals two Tren^TIPS^–uranium units bridged by a side-on μ-η^2^:η^2^-S_2_ moiety. Each uranium centre is six-coordinate, ligated by the four N atoms of a tetradentate Tren^TIPS^ ligand and two sulfur atoms and adopts a distorted octahedral geometry. The U–N_amide_ and U–N_amine_ bond distances in **4** of 2.264(3) (av.) and 2.709(3) Å are typical for uranium(iv)–N_amide_ and –N_amine_ bonds,^45^ respectively, and compare favourably to those in **2**. The U–S bond distances of 2.8666(12) and 2.9280(15) Å are statistically distinct and roughly lie within range of the corresponding bond lengths in uranium(iv) persulfide complexes [2.7062(16)–2.9228(15) Å] which reflects the large steric demands of Tren^TIPS^, and the S1–S1A bond distance of 2.1041(18) Å is identical to reported S–S persulfide single bond distances.^42,43^ The solid state structure of **4** adopts a symmetrical U_2_S_2_ core and possesses a crystallographic inversion centre at the midpoint of the S–S bond. As a whole the structural data support the assignment of a persulfide (S_2_^2–^) unit bridged between two uranium(iv) centres in **4**.

The ^1^H NMR spectrum of **4** features four paramagnetically shifted resonances in the range –37 to +33 ppm, consistent with an approximate threefold-symmetry of the Tren^TIPS^ ligands. The room temperature solution magnetic moment (Evans method) of **4** in pyridine of 3.89 *μ*_B_ is consistent with the presence of two 5f^2^ uranium(iv) centres and compares to a value of 3.74 *μ*_B_ for the dinuclear bridged Tren^TIPS^–uranium(iv) sulfide complex **2**. Variable temperature magnetometry on a solid sample of **4** revealed a *μ*_eff_ value of 3.76 *μ*_B_ at 300 K (Fig. S2†), which is in excellent agreement with that determined from NMR. This value decreases steadily to 3.32 *μ*_B_ at 100 K at which point the value of *μ*_eff_ drops off more sharply to 1.46 *μ*_B_ at 1.8 K.

The isolation of complex **4** (and not **2**) from **1** with S_8_ demonstrates the sensitive nature of the reactivity profile of **1** with sulfur containing reagents, a feature that has also been observed in the reactivity of the related uranium(iii)–triamide complex [U{N(SiMe_3_)_2_}_3_].^42^ This was further investigated by treating **4** with KC_8_ in an attempt to produce a Tren–uranium complex with a terminal S functionality that could benefit from additional stabilisation from coordinated K^+^ ions. However, **4** reacts with one equivalent of KC_8_ (Scheme 1) to afford a brown mixture from which a gray solid containing graphite and potassium sulfide, K_2_S, was separated by filtration. A crop of orange crystals was isolated from the filtrate after cooling to 5 °C, which was identified as **2** by a crystallographic unit cell check and ^1^H NMR spectroscopy. The very low yield of **2** from this reaction (7%) highlights the consistently poor crystalline yields of **2** due to the inherent sensitivity and lability of U–S and E–E bonds,^44,54,55^ and also the thermodynamic favourability of U–S–U formation, given that **2** is the only product of note from this reaction.

**Fig. 4:**
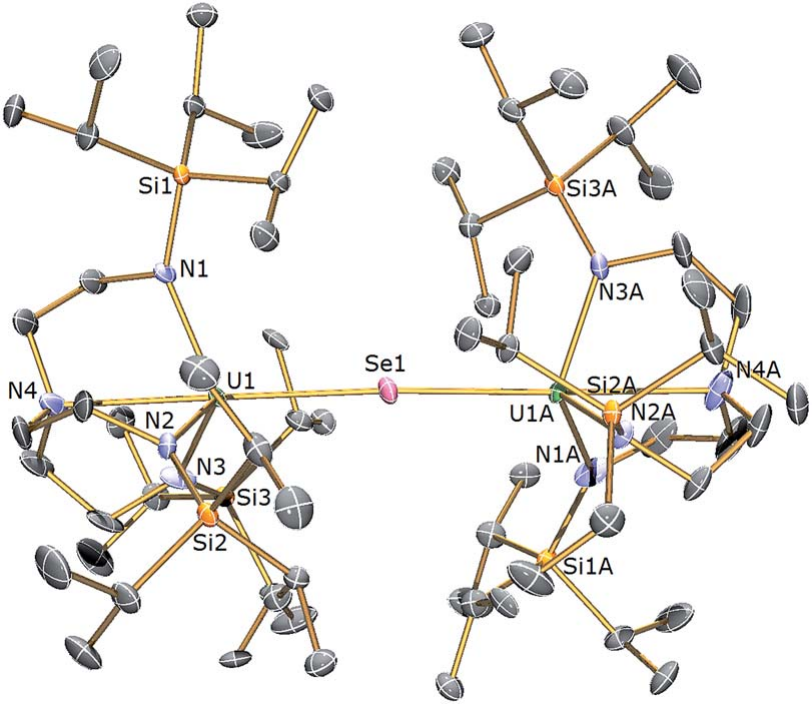
Molecular structure of **5**. Displacement ellipsoids set at 40% probability. H atoms and lattice solvent molecules are omitted for clarity. Selected bond distances (Å) and angles (°): U1–Se1 2.8100(2), U1–N1 2.254(3), U1–N2 2.259(4), U1–N3 2.256(3), U1–N4 2.683(4), U1–Se1–U1 175.58(3), N4–U1–Se1 178.87(11).

**Fig. 5:**
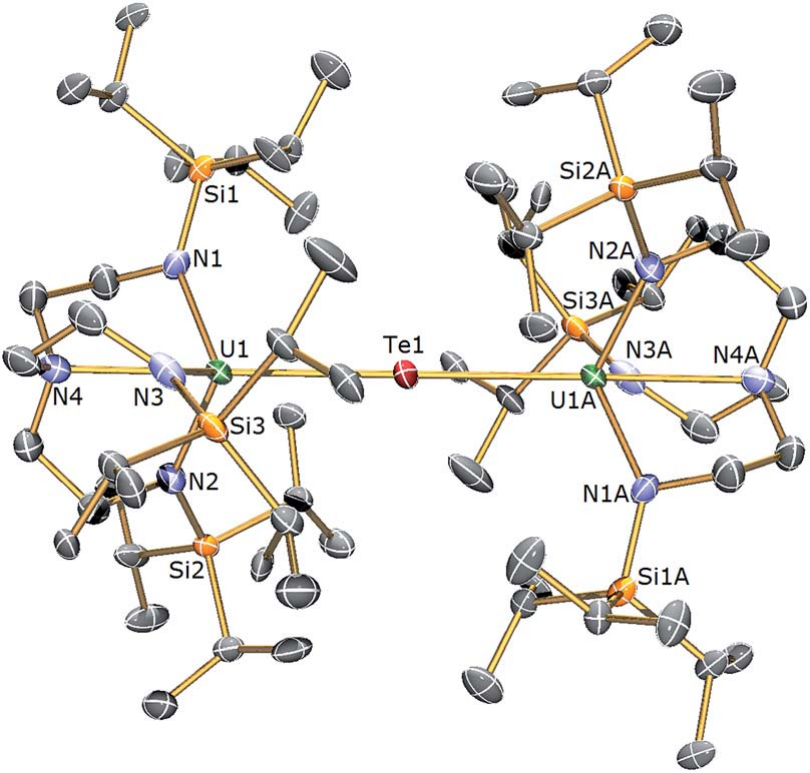
Molecular structure of **6**. Displacement ellipsoids set at 40% probability. H atoms and lattice solvent molecules are omitted for clarity. Selected bond distances (Å) and angles (°): U1–Te1 3.0807(3), U1A–Te1 3.0807(4), U1–N1 2.225(9), U1–N2 2.205(10), U1–N3 2.211(10), U1–N4 2.709(12), U1–Te1–U1 180.0, N4–U1–Te1 177.5(3).

The reactivity of **3** towards the heavier chalcogens was then explored. Treatment of **3** with one equivalent of either elemental selenium or tellurium afforded, after work-up and crystallisation, dark orange or red crystals, respectively, suitable for single crystal XRD studies. These revealed the molecular structures of the products to be [{U(Tren^TIPS^)}_2_(μ-Se)] (**5**) and [{U(Tren^TIPS^)}_2_(μ-Te)] (**6**) are illustrated in Fig. 4 and 5 with selected bond lengths and angles.

The solid state structures of **5** and **6** are similar to **2**, with two Tren^TIPS^–uranium units bridged by Se or Te atoms in **5** and **6**, respectively. The uranium ions are five-coordinate and adopt distorted trigonal bipyramidal geometries with the chalcogenide atom located *trans* to the amine centres [N4–U1–Se1 = 178.87(11)° for **5**, N4–U1–Te1 = 177.5(3)° for **6**]. The U–N_amide_ and U–N_amine_ bond distances of 2.256(4) (av.) and 2.683(4) Å in **5** and 2.214(10) and 2.709(12) Å in **6**, respectively, are typical for uranium(iv)–N_amide_ and –N_amine_ bonds and support a U^IV^–E–U^IV^ formulation.^45^ The U–Se bond distances in **5** of 2.8101(20) Å and the U–Te bond distances in **6** of 3.0807(3) Å compare well to the sum of the covalent single bond radii (U–Se: 2.86; U–Te: 3.06 Å)^46^ and to reported U^IV^–E–U^IV^ bond distances generally.^14,42^ The U–E bond lengths in **2**, **5** and **6** show the expected increase in the order U–S < U–Se < U–Te, each being successively longer by ∼0.2 Å and the U–E–U bond angles are expectedly linear or near-linear [U1–Se1–U1 = 175.56(2)° for **5**, U1–Te1–U1 = 180.0° for **6**].

The ^1^H NMR spectrum of **5** features seven paramagnetically shifted resonances in the range –31 to +10 ppm, which, by analogy to **2**, is suggested to be due to the eclipsed orientation of the Tren^TIPS^ ligands on the NMR timescale. The ^1^H NMR spectrum of **6**, however, features only three broad resonances, which by contrast to **2** and **5** is ascribed to the staggered orientation of the Tren^TIPS^ ligands observed in the solid state structure being maintained in solution on the NMR timescale. The room temperature solution magnetic moments (Evans method) of **5** and **6** in benzene of 3.88 and 4.10 *μ*_B_ are each consistent with the presence of two 5f^2^ uranium(iv) centres and are similar to that measured for **2**. These data are reflected in variable temperature magnetometry experiments on powdered **5** and **6**, which reveal magnetic moments of 3.5 and 3.75 *μ*_B_ at 298 K, respectively, that fall monotonously reaching values of ∼0.7 *μ*_B_ at 1.8 K and tending to zero (Fig. 8). Notably, the magnetic data for **2**, **5**, and **6**, whether powdered or in solution, exhibit magnetic moments that are ordered **6** > **5** > **2**.

### Electronic structure and magnetic analysis of **2**, **5**, and **6**

**Fig. 6:**
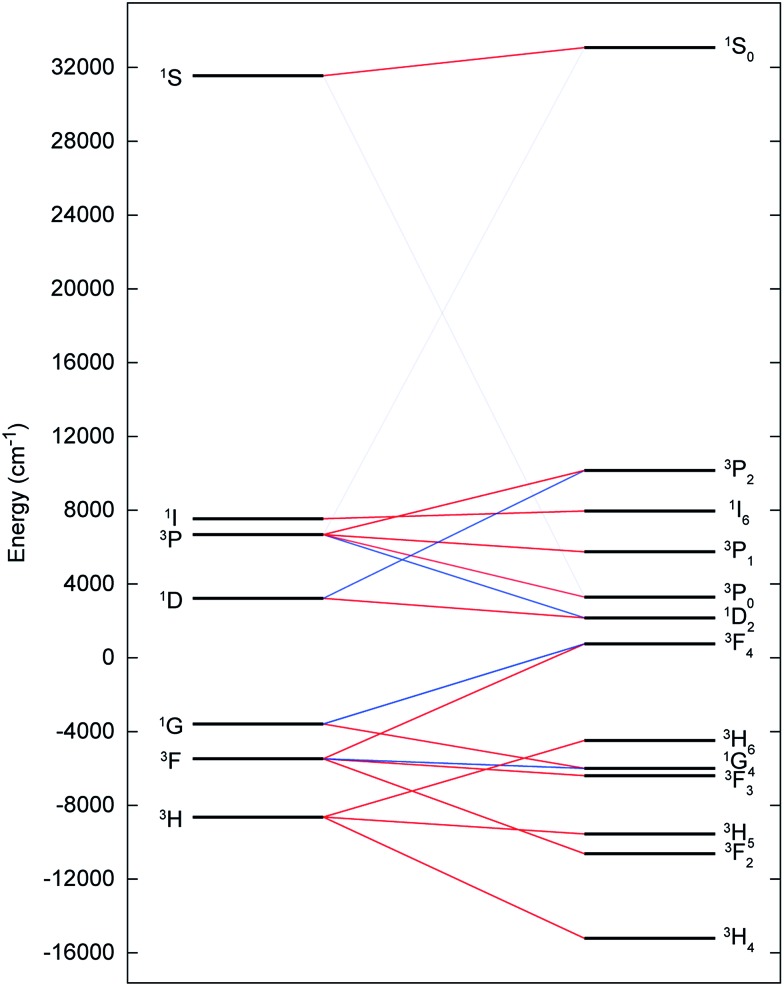
CASSCF-SO-calculated electronic states for the [Rn]5f^2^ configuration of the gas-phase U^IV^ ion under the influence of IER (left) and both IER and SOC (right). Red lines show parentage of SOC multiplets and blue lines show mixing between different electronic terms due to SOC; opacity of these lines reflect the extent of the mixing. The SOC mixing occurs between states with the same *J* quantum number and is stronger when the parent terms are closer in energy, hence the ^1^S_0_ ↔ ^3^P_0_ mixing is much weaker than the ^3^F_4_ ↔ ^1^G_4_ mixing, for example.

**Fig. 7:**
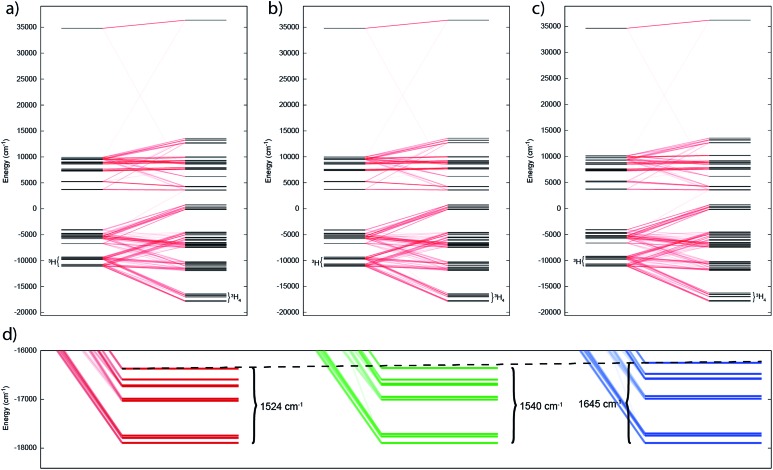
Electronic states for (a) **2**, (b) **5** and (c) **6** under the influence of IER (inner left) and both IER and SOC (inner right). Red lines show parentage of SOC multiplets; opacity of these lines reflect the extent of the mixing. (d) Close up view of ground ^3^H_4_ manifold for **2**, **5** and **6** (left to right) with span of the term given. Dotted guide line shows increase in span from left to right.

The electronic ground state of uranium(iv) is well defined by the [Rn]5f^2^ configuration, with the next electronic configuration [Rn]5f^1^6d^1^ lying at *ca.* 100 000 cm^–1^. Following the Russell–Saunders (RS) scheme for angular momentum coupling, IER splits the [Rn]5f^2^ configuration into terms, the ground one being ^3^H given by Hund's rules (Fig. 6, left). SOC then splits these terms into total angular momentum states *J* = |*L* – *S*|, |*L* – *S*| + 1, …, *L* + *S* – 1, *L* + *S* (Fig. 6, right), with the ^3^H_4_ SO multiplet lying lowest *ca.* 5000 cm^–1^ below the ^3^F_2_ SO multiplet arising from the first excited ^3^F term. When the uranium(iv) ion is incorporated into a molecular complex, the electronic states are split owing to the formation of molecular orbitals (MO); for transition metal complexes this is often described as the effect of the CF and we use the same nomenclature here. For all three complexes, **2**, **5** and **6**, and despite the large radial extent of the 5f orbitals compared to 4f orbitals, the CF generated by the Tren^TIPS^ and E ligands is rather small compared to both the IER and SOC (Fig. 7 *cf.* Fig. 6). Thus, the low-lying electronic states of each uranium(iv) ion in complexes **2**, **5** and **6** are well-described by the ^3^H_4_ SOC multiplet split by the CF; this is the familiar case for lanthanide ions, and thus uranium(iv) resembles the [Xe]4f^2^ Pr^III^ ion in this case.

In such cases where SOC > CF, it is more appropriate to consider the action of the CF on the ground *J* manifold. Thus, for ligand environments with trigonal bipyramidal symmetry such as these, the *J* = 4 state will be split into three singlets and three doublets. However as uranium(iv) is a non-Kramers ion (even number of unpaired electrons), the CF can entirely remove the degeneracy of the ^3^H_4_ multiplet, resulting in 9 singlet states in low symmetry; importantly, these singlets are highly anisotropic and will be affected by a magnetic field and thus uranium(iv) species are not diamagnetic at low temperature, and rather show temperature independent paramagnetism (TIP). Indeed, complete active space self-consistent field spin–orbit (CASSCF-SO) calculations (see Experimental Details) show that **2**, **5** and **6** all possess singlet ground states (all of which have dominant contributions from the *m*_*J*_ = 0 state of 74, 85 and 55%, respectively, quantised along the pseudo-*C*_3_ axis), where the *J* = 4 multiplet is split over 1500–1600 cm^–1^ (Tables S1–S3†). While the splitting of the ground multiplet for the three complexes is broadly quite similar, there is a systematic increase in CF splitting from **2**, **5** and **6**, despite the systematic lengthening of the U–E bond, suggesting that the donor strength increases as S < Se < Te. Trends are also observed in the LoProp charges on the bridging chalcogenide atom and in the percentage of the active space made up of E-based atomic orbitals (AOs) (Table S4†), suggesting that the increase in field strength is due to increased charge accumulation on the chalcogenide atom whilst the covalency may actually be decreasing as S > Se > Te.

**Fig. 8:**
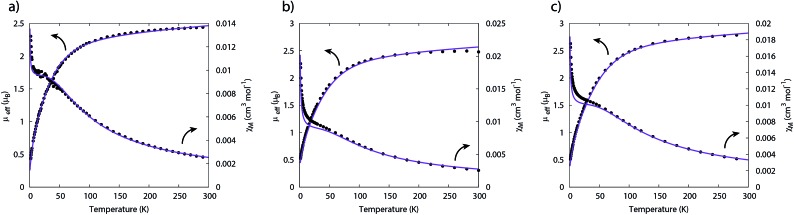
Temperature dependence of the magnetic moment for (a) **2**, (b) **5** and (c) **6** (per uranium) measured in a 0.1 T field. Solid purple lines are fits with Hamiltonian eqn (3) with CF parameters from CASSCF-SO (Table S5†) and those in Table 1.

The temperature dependence of the magnetic moment of polycrystalline **2**, **5** and **6** are all broadly similar (Fig. 8); *μ*_eff_ has values of 2.46–2.78 *μ*_B_ (*χT* = 0.75–0.96 cm^3^ mol^–1^ K) per uranium at room temperature, in good agreement with that expected for uranium(iv),^47^ that decrease slowly upon cooling until 100 K when they drop more rapidly reaching 0.43–0.53 *μ*_B_ (*χT* = 0.02–0.04 cm^3^ mol^–1^ K) per uranium at 1.8 K (Fig. 8). The magnetic susceptibilities plateau at around 50 K with values of *ca.* 0.01 cm^3^ mol^–1^ for all three complexes, before increasing rapidly below 10 K. The observation of such plateaus is direct evidence of a singlet ground state, in agreement with the CASSCF-SO calculations; if the ground state were a pseudo-doublet then the susceptibility would be Curie-like and behave as *χ* ∝ 1/*T* at all temperatures.

Plateaus such as these are but one of three experimentally observed low temperature profiles for mono and dimetallic uranium(iv) species. In addition to plateaus,^48,56^ continual Curie-like rising of the susceptibility^14,27,48,52^ and Néel-type maxima^14,15^ are also observed. Given that *χ vs. T* profiles are sometimes employed to directly infer the presence or absence of magnetic interactions between uranium ions,^14,15,27^ it would be helpful to have a guide to aid in such interpretations; hence, we seek to simulate a set of magnetic traces for mono and dimetallic U^IV^ species, to aid in the identification and characterisation of magnetic interactions. While the CF will be different for each molecule, we employ a simplified CF model to highlight the differences between singlet and (pseudo-)doublet ground states for individual uranium(iv) centres. Thus our model consists of a single axial CF term for each uranium centre, along with an exchange interaction of the Lines type (Hamiltonian eqn (1) and (2)).^57^ We performed our simulations with PHI^58^ using the |*J* = 4, *m*_*J*_ basis for each uranium(iv) centre, where *B*02 are the axial CF parameters, *Ô*02 are the Stevens operators, *J* is the Lines exchange parameter, *g*_*J*_ is the Landé *g*-factor, and the exchange term is treated using a Clebsch–Gordan decomposition. We choose our CF parameter to be *B*02 = ±30 cm^–1^ (positive for a singlet *m*_*J*_ = 0 ground state and negative for a doublet *m*_*J*_ = ±4 ground state), chosen to generate CF splitting on the order of magnitude calculated for **2**, **5** and **6**, and fix *g*_*J*_ = 0.8 from the free-ion ^3^H_4_ SO multiplet. The magnetic susceptibility is simulated from 1.8–300 K in a field of 0.1 T using the expression *χ* ≈ *M*/*B* in order to match the most common experimental conditions.

1 *Ĥ* = *B*02*Ô*02 + *μ*_B_
*g*_*J*_
*Ĵ*·*B*

2

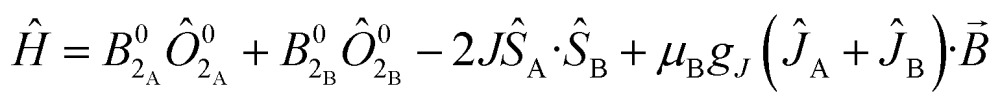




For a monometallic species where inter-uranium magnetic interactions cannot occur (excluding through-space dipolar couplings that are expected to be negligible for magnetic measurements, though detectable with EPR^5^), a plateau in the susceptibility must arise from isolation of a singlet ground state due to the CF (Fig. S3a†); indeed this feature has been observed previously for monometallic uranium(iv) complexes.^56,59–62^ In such cases, any further increase at the lowest temperatures can be confidently attributed to a small paramagnetic impurity which can be very difficult to avoid when dealing with air-sensitive samples – even 1% impurity can easily be seen by sensitive magnetic measurements (Fig. S5†). If on the other hand the susceptibility for a monometallic uranium(iv) species appears Curie-like at all temperatures, this is indicative of a (pseudo-)doublet ground state (Fig. S3b†).

When considering the presence of a magnetic interaction for dimetallic species the picture is much less clear (Fig. S3, S4 and S6–S8†). In fact, there are only two cases where the magnetic trace can unequivocally define magnetic interactions under visual inspection:

(1) *χ vs. T* shows a Néel-type maximum at low temperature, corresponding to a pair of uranium(iv) ions with (pseudo-)doublet ground states with an antiferromagnetic interaction;

(2) *μ*_eff_ or *χT vs. T* shows an increase at low temperature, corresponding to a pair of uranium(iv) ions with (pseudo-)doublet ground states with a ferromagnetic interaction.

A trace of any other type could easily be assigned to a number of different situations, and thus in such situations it is inappropriate to assign the presence of a magnetic interaction on this data alone. Furthermore, it is also possible that a small yet not insignificant magnetic impurity may be obscuring the true low temperature behaviour; this is particularly pertinent for uranium magnetic data given the wide range of room temperature magnetic moments reported and overlaps between oxidations states.^47^

Therefore, using these guidelines, the magnetic data for compounds **2**, **5** and **6** do not indicate significant magnetic interactions, though they cannot be absolutely ruled out. As both sites are crystallographically equivalent and the CASSCF-SO calculations suggest a well-isolated singlet for the individual U^IV^ ions, we interpret the plateaus in *χ* at low temperatures as confirmation of this feature, while the rise below 10 K is attributed to a small paramagnetic impurity. The CF parameters for the ^3^H_4_ SO multiplet obtained directly from the CASSCF-SO calculations (Table S5†) lead to simulations of the magnetic susceptibility that have extremely similar profiles to the magnetic data per uranium for **2**, **5** and **6** (Fig. S9–S11†), however the absolute magnitude is incorrect. These simulations employ the free-ion |*J* = 4, *m*_*J*_ basis, while the true magnetic orbitals are MOs with non-negligible ligand character; a common approach to account for this is to include an orbital reduction parameter, *κ*.^5,63^ We include this parameter by allowing *g*_*J*_ to be reduced from 0.80 (indeed this is also suggested by CASSCF-SO, Table S4†); using *g*_*J*_ as a variable, and incorporating a small *S* = 1 Curie-like impurity, an excellent fit of the experimental data is obtained (Fig. 8, S12 and S13,† and Table 1). By re-arranging the equation for the Landé *g*-factor,^64^ the resulting *g*_*J*_ values for uranium(iv) can be re-cast as orbital reduction parameters with the expression *κ* = 2/6 + 5*g*_*J*_/6; these are found to be between 0.85 and 0.92, which is of a similar magnitude to those determined for the [(Tren^TIPS^)U^V^(N)]^–^ anion of between 0.88 and 0.97.^5^ The trend of decreasing covalency across the series as suggested by the percentage of E-based AOs in the CASSCF active space as S > Se > Te is also reflected experimentally by the increase in *κ* in the same sense; however, we note that *κ,* whilst accommodating the effects of covalency on the magnetic properties, does not provide an unequivocal measure of covalency and therefore should not be compared between different molecular series where other factors, such as oxidation state and CF, will play a major role in the electronic structure and bonding.
3

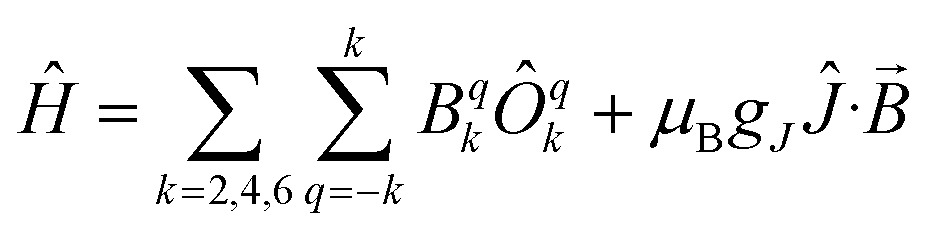




**Table 1:** Parameters derived from fitting magnetic data for complexes **2**, **5** and **6**

Parameter	**2**	**5**	**6**
*g*	0.621	0.644	0.705
*S* = 1 impurity (%)	0.25	1.4	0.86
*κ*	0.851	0.870	0.921

## Conclusions

To conclude, we have prepared three diuranium μ-chalcogenide complexes along with one persulfide. Complexes with U–E–U (E = Se, Te) cores are consistently formed, but for sulfur U–S–U and U(S_2_)U could be selectively obtained. The essentially linear U–E–U cores provide an opportunity to study the magnetism of these linkages where plots of *χ vs. T* present shoulders that could be interpreted as evidence of uranium–uranium magnetic exchange. However, a detailed study of the electronic structure of these uranium complexes reveals that the magnetic properties of these systems can be simply correlated to single-ion CF effects that vary as the nature of the chalcogen varies. Elucidating the presence of magnetic coupling between actinide ions is an area fraught with difficulty and ambiguity, and although a maximum in *χ vs. T* is usually good evidence for magnetic exchange, there are numerous examples where it would be tempting to suggest magnetic exchange occurs (*i.e.* chemically plausible scenarios) when in reality the behaviour is instead actually the manifestation of CF effects. The present study highlights this caveat and places the assignment of CF effects, rather than magnetic exchange, on a firmer footing thus enabling assignments to be made with more confidence more broadly.

## Experimental

### General

All manipulations were carried out under an inert atmosphere of dry nitrogen utilising standard Schlenk techniques, or an MBraun UniLab glovebox operating under an atmosphere of dry nitrogen with H_2_O and O_2_ <0.1 ppm. Complex **1** and KC_8_ were prepared according to published procedures.^28,65^ HMPA, Ph_3_PS, sulfur, selenium and tellurium were purchased from Sigma-Aldrich and either vacuum distilled (HMPA) or dried *in vacuo* (Ph_3_PS, S_8_, Se and Te) and stored under nitrogen. THF, hexane and toluene were dried by passage through activated alumina, degassed prior to use and stored over activated 4 Å molecular sieves (THF) or a potassium mirror (hexane, toluene). Diethylether was distilled from sodium–potassium alloy and stored over a potassium mirror. Deuterated NMR solvents were purchased from Goss Scientific Ltd. C_6_D_6_ was distilled from a potassium mirror, pyridine-*d*_5_ was distilled from CaH_2_ and both were degassed by three freeze–pump–thaw cycles and stored under nitrogen. Nujol was purchased from Sigma-Aldrich and degassed prior to use.

^1^H, ^29^Si and ^31^P NMR spectra were recorded on a Bruker AV400 or AV(III)400 spectrometer [operating at 400.2 (^1^H), 79.5 (^29^Si) and 162.0 (^31^P) MHz, respectively]. Chemical shifts (*δ*) are quoted in ppm and are relative to external TMS (^1^H, ^29^Si) or 85% H_3_PO_4_ (^31^P). Samples were prepared in the glovebox and placed in J. Young PTFE 5 mm screw-topped borosilicate NMR tubes. Capillary tube inserts for Evans method magnetic measurements were sealed under nitrogen using a volumetric ratio of 7 : 3 of non-deuterated : deuterated solvent. The relatively low solubility of the dinuclear complexes **2** and **4–6** precluded the acquisition of ^29^Si NMR spectra for those complexes. Crystals were examined on a Bruker APEX CCD area detector diffractometer or an Oxford Diffraction Ltd. SuperNova Atlas CCD diffractometer. FTIR spectra were recorded on a Bruker Tensor 27 spectrometer and samples were prepared in the glovebox as Nujol mulls between KBr discs. Elemental analyses were performed by Dr Tong Liu (University of Nottingham). Variable-temperature SQUID magnetic measurements were performed in an applied DC field of 0.1 T on Quantum Design MPMS-XL 5 or XL 7 SQUID magnetometers using doubly-recrystallised powder samples. Samples were checked for purity before and after use and data reproducibility was carefully checked. Care was taken to ensure complete thermalisation of the sample before each data point was measured. Diamagnetic corrections (*χ*_D_) were applied using tabulated Pascal constants and measurements were corrected for the effect of the blank sample holders.

### Computational

CASSCF-SO calculations were performed using MOLCAS 8.0 (ref. 66) on the crystal structures of **2**, **5** and **6** without optimisation. The single-site properties for the structurally unique uranium site in each molecule were determined with the other site computationally substituted for the diamagnetic thorium(iv). Basis sets of the ANO-RCC library were employed^67,68^ with VTZP quality for the uranium atom, VDZP quality for the first coordination sphere atoms, and VDZ quality for all other atoms. All 21 triplets and 28 singlets were employed in the orbital optimisation and configuration interaction step, as well as being subsequently mixed by SO coupling. CF parameters were extracted using SINGLE_ANISO.^69^

### Preparation of [{U(Tren^TIPS^)}_2_(μ-S)] (**2**)

#### Method A

A solution of **1** (0.85 g, 1.0 mmol) in toluene (10 ml) was added slowly to a cold (–78 °C) stirring solution of triphenylphosphine sulfide (0.29 g, 1.0 mmol) in toluene (10 ml). The mixture was allowed to warm to room temperature whilst stirring over 16 h, affording a yellow precipitate. The solid was collected by filtration, washed with hexanes (3 × 2 ml) and dried *in vacuo*. The solid was recrystallised from hot (50 °C) THF at 5 °C which yielded yellow crystals that were isolated by filtration, washed with toluene (3 × 2 ml) and dried for 30 minutes. Yield: 0.33 g, 35%.

#### Method B

Toluene (10 ml) was added slowly to a stirring mixture of **3** (0.51 g, 0.5 mmol) and triphenylphosphine sulfide (0.15 g, 0.5 mmol) at –78 °C. The mixture was allowed to warm to room temperature whilst stirring over 16 h, which afforded a yellow precipitate. The solid was collected by filtration, washed with hexanes (3 × 2 ml) and dried *in vacuo*. The solid was recrystallised at 50 °C from hot THF which yielded yellow crystals of **2** which were isolated by filtration, washed with toluene (3 × 2 ml) and dried for 30 minutes. Yield: 0.15 g, 33%.

#### Method C

THF (15 ml) was added to a cold (–78 °C) stirring mixture of **4** (0.10 g, 57 μmol) and KC_8_ (15 mg, 113 μmol) and the resulting brown suspension allowed to warm to ambient temperature and stirred at this temperature for 16 h to afford a dark yellow solution and a black solid. The solution was filtered and solvent was removed *in vacuo* to afford a yellow solid that was dissolved in warm (60 °C) toluene (5 ml) and reduced in volume to *ca.* 0.5 ml. Crystalline material was obtained by storage of this solution at room temperature overnight and the yellow crystals obtained were isolated by filtration, washed with toluene (3 × 2 ml) and dried for 30 minutes. Yield: 7 mg (7%). ^1^H NMR (C_6_D_6_, 298 K): *δ* –21.73 (s, 6H, C*H*_2_), –10.87 (s, 6H, C*H*_2_), –5.82 (s, 6H, C*H*_2_), –3.26 (s, 18H, C*H*(CH_3_)_2_), –0.07 (s, 6H, C*H*_2_), 4.49 (s, 54H, CH(C*H*_3_)_2_), 6.96 (s, 54H, CH(C*H*_3_)_2_). *μ*_eff_ (Evans method, C_6_D_6_, 298 K): 3.74 *μ*_B_. FTIR *ν*/cm^–1^ (Nujol): 1378 (m), 1338 (m), 1142 (m), 1105 (m), 1040 (s), 1013 (s), 934 (s), 914 (w), 891 (m), 877 (m), 807 (s), 741 (s), 676 (w), 660 (w), 633 (m). Anal. calc. for C_66_H_150_N_8_SSi_6_U_2_·C_4_H_8_O: C, 46.59; H, 8.82; N, 6.21. Found: C, 46.28; H, 8.82; N, 6.70.

### Preparation of [U(Tren^TIPS^)(HMPA)] (**3**)

A solution of HMPA (0.09 g, 0.5 mmol) in hexanes (2 ml) was added dropwise to a dark blue stirring solution of **1** (0.42 g, 0.5 mmol) in toluene (10 ml) at –78 °C. The dark green solution produced was allowed to warm to room temperature whilst stirring over 16 h. The resulting precipitate was isolated by filtration and washed with hexanes (2 × 5 ml) to yield **3** as a very dark green solid. Complex **3** was recrystallised from a saturated solution of hexanes at 5 °C. Yield: 0.40 g, 78%. ^1^H NMR (C_6_D_6_, 298 K): *δ* –3.95 (s, 6H, C*H*_2_), 1.10 (s, 18H, N(C*H*_3_)_2_), 2.28 (s, 54H, CH(C*H*_3_)_2_), 2.76 (s, 9H, C*H*(CH_3_)_2_), 5.60 (s, 6H, C*H*_2_). ^29^Si{^1^H} NMR (C_6_D_6_, 298 K): *δ* –5.47. ^31^P{^1^H} NMR (C_6_D_6_, 298 K): 90.02. *μ*_eff_ (Evans method, C_6_D_6_, 298 K): 3.02 *μ*_B_. FTIR *ν*/cm^–1^ (Nujol): 1300 (w), 1261 (m), 1193 (w), 1119 (s), 1066 (s), 1027 (s), 992 (s), 936 (m), 883 (m), 801 (s), 746 (s), 670 (w), 629 (w). Anal. calc'd for C_39_H_93_N_7_OPSi_3_U: C, 45.50; H, 9.11; N, 9.52. Found: C, 44.75; H, 9.05; N, 9.36.

### Preparation of [{U(Tren^TIPS^)}_2_(μ-η^2^:η^2^-S_2_)] (**4**)

A green-black solution of **3** (0.61 g, 0.6 mmol) in toluene (10 ml) was added dropwise to a cold stirring (–78 °C) suspension of S_8_ (19 mg, 75 μmol) in diethyl ether (10 ml) over ten minutes. The resulting brown-black mixture was allowed to warm to ambient temperature over 1 hour after which time a colour change to orange-brown was observed accompanied by the consumption of any residual sulfur. After stirring at ambient temperature for a further 1 h, removal of solvent *in vacuo* and suspension of the residue in hexanes (10 ml) afforded an orange-yellow solid that was isolated by filtration, washed with hexanes (3 × 2 ml) and dried for 30 minutes. This product is essentially pure but may be recrystallised from a saturated toluene solution at –30 °C, although crystalline yields are low. Yield: solid 0.18 g (35%) or crystalline 50 mg (10%). ^1^H NMR (pyridine-*d*_5_, 298 K): *δ* –36.97 (s, 12H, C*H*_2_), –6.18 (s, 108H, CH(*CH*_3_)_2_), –5.52 (s, 18H, C*H*(CH_3_)_2_), 32.63 (s, 12H, C*H*_2_). *μ*_eff_ (Evans method, pyridine-*d*_5_, 298 K): 3.89 *μ*_B_. FTIR (Nujol): *ν̃* 1307 (w), 1261 (m), 1141 (m), 1095 (s), 1030 (s), 934 (w), 917 (w), 880 (w), 802 (s), 723 (s), 675 (w), 631 (w), 609 (w) cm^–1^. Anal. calc'd for C_66_H_150_N_8_S_2_Si_6_U_2_: C 44.81%; H 8.60%; N 6.35%. Found: C 44.68%; H 8.56%; N 6.39%.

### Preparation of [{U(Tren^TIPS^)}_2_(μ-Se)] (**5**)

A green-black solution of **3** (0.61 g, 0.6 mmol) in toluene (5 ml) was added dropwise to a cold stirring (–78 °C) suspension of selenium (47 mg, 0.6 mmol) in diethyl ether (10 ml) over ten minutes. The resulting dark brown-black mixture was allowed to warm to ambient temperature over 1 h after which time a colour change to green-black was observed. A further gradual colour change to yellow-brown was observed by stirring the mixture at ambient temperature for a further 16 h. Removal of solvent *in vacuo* and suspension of the residue in toluene (10 ml) afforded an orange-yellow solid that was isolated by filtration, washed with hexane (3 × 2 ml) and dried for 30 minutes. This product is essentially pure but could be crystallised from a saturated toluene solution at –30 °C. Yield: 0.16 g (31%). ^1^H NMR (C_6_D_6_, 298 K): *δ* –30.76 (s, 6H, C*H*_2_), –18.84 (s, 6H, C*H*_2_), –7.85 (s, 6H, C*H*_2_), –1.38 (s, 6H, C*H*_2_), 3.89 (s, 18H, C*H*(CH_3_)_2_), 5.57 (s, 54H, CH(C*H*_3_)_2_), 9.18 (s, 54H, CH(C*H*_3_)_2_). *μ*_eff_ (Evans method, C_6_D_6_, 298 K): 3.88 *μ*_B_. FTIR (Nujol): *ν̃* 1305 (m), 1262 (m), 1143 (m), 1076 (s), 1038 (s), 1016 (s), 932 (m), 887 (m), 847 (w), 803 (m), 734 (s), 724 (s), 675 (w), 659 (w), 634 (w), 588 (w), 569, 554 (w) cm^–1^. Anal. calc'd for C_66_H_150_N_8_SeSi_6_U_2_·1.35C_7_H_8_: C 47.60%; H 8.51%; N 5.89%. Found: C 47.59%; H 8.57%; N 5.89%.

### Preparation of [{U(Tren^TIPS^)}_2_(μ-Te)] (**6**)

A green-black solution of **3** (0.61 g, 0.6 mmol) in toluene (5 ml) was added dropwise to a cold stirring (–78 °C) suspension of tellurium (76 mg, 0.6 mmol) in diethyl ether (10 ml) over ten minutes. The resulting dark brown-black mixture was allowed to warm to ambient temperature over 1 h and stirred at ambient temperature for a further 16 h over which time a gradual colour change to brown was observed along with precipitation of a red solid. The red solid was isolated by filtration, washed with THF (3 × 2 ml), extracted into 10 ml hot (60 °C) THF and filtered away from any insoluble tellurium residues. Removal of solvent *in vacuo* afforded a red solid that was essentially pure but could be crystallised from 2 ml of a saturated THF solution at 5 °C. Yield: 0.17 g (32%). ^1^H NMR (C_6_D_6_, 298 K): *δ* –6.13 (s, 12H, C*H*_2_), 4.42 (s, 18H, C*H*(CH_3_)_2_), 8.76 (s, 120H, CH(C*H*_3_)_2_ + C*H*_2_). *μ*_eff_ (Evans method, C_6_D_6_, 298 K): 4.10 *μ*_B_. FTIR (Nujol): *ν̃* 1297 (w), 1272 (w), 1259 (w), 1140 (w), 1129 (w), 1114 (w), 1063 (w), 1048 (w), 1035 (m), 1011 (m), 969 (w), 931 (s), 909 (w), 886 (s), 804 (m), 731 (s), 676 (m), 658 (w), 634 (m), 571 (w), 551 (w), 519 (w), 445 (w) cm^–1^. Anal. calc'd for C_66_H_150_N_8_Si_6_TeU_2_: C 43.35%; H 8.28%; N 6.13%. Found: C 43.33%; H 8.34%; N 6.28%.
