# Supplementary material for: Assessing crystal field and magnetic interactions in diuranium-μ-chalcogenide triamidoamine complexes with UIV–E–UIV cores (E = S, Se, Te): implications for determining the presence or absence of actinide–actinide magnetic exchange
Source: Chem Sci. 2017 Jul 5;8(9):6207–17. doi: 10.1039/c7sc01998j (PMC5628351; doi:10.1039/c7sc01998j)
Supplement: Supplementary file 1 [file SC-008-C7SC01998J-s001.pdf]

Electronic Supplementary Material (ESI) for Chemical Science.
This journal is © The Royal Society of Chemistry 2017

- Electronic Supplementary Information -

Assessing Crystal Field and Magnetic Interactions in Diuranium-μ-Chalcogenide Triamidoamine Complexes With U^{IV}-E-U^{IV} Cores (E = S, Se, Te): Implications for Determining the Presence or Absence of Actinide-Actinide Magnetic Exchange

Benedict M. Gardner,[1] David M. King,[2] Floriana Tuna,[1] Ashley J. Wooles,[1] Nicholas F. Chilton,[1*] and Stephen T. Liddle[1*]

[1] School of Chemistry, The University of Manchester, Oxford Road, Manchester, M13 9PL, UK. [2] School of Chemistry, The University of Nottingham, University Park, Nottingham, NG7 2RD, UK. *Email: steve.liddle@manchester.ac.uk; nicholas.chilton@manchester.ac.uk.

Supplementary Figures and Tables

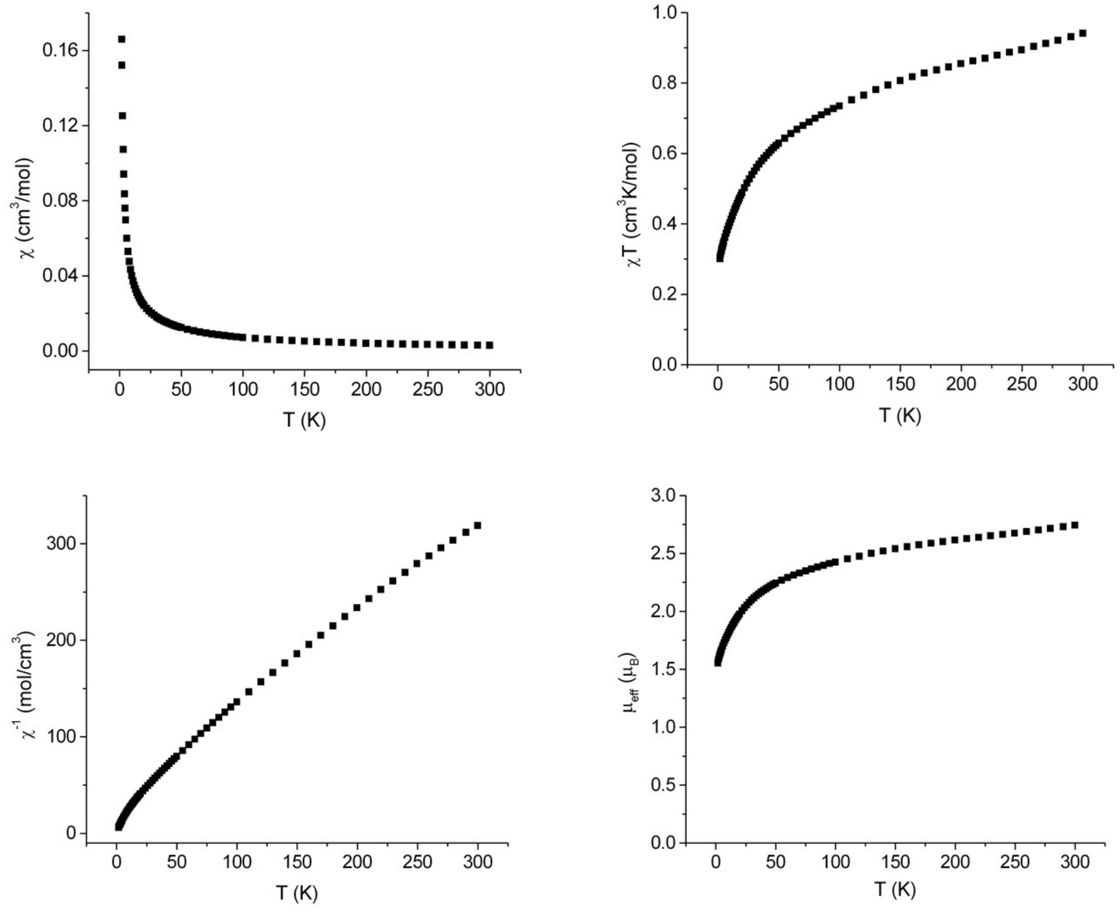

Figure S1. Magnetic data for **3** recorded in a 1 kG field.

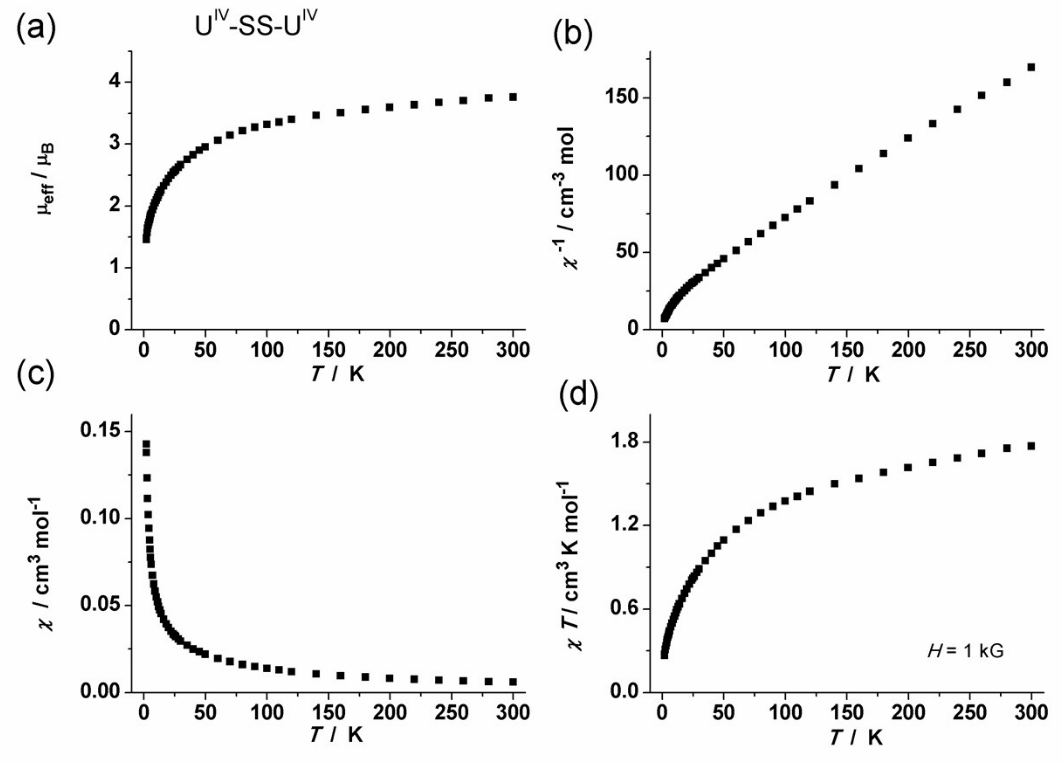

Figure S2. Magnetic data for **4**, recorded in a 1 kG field.

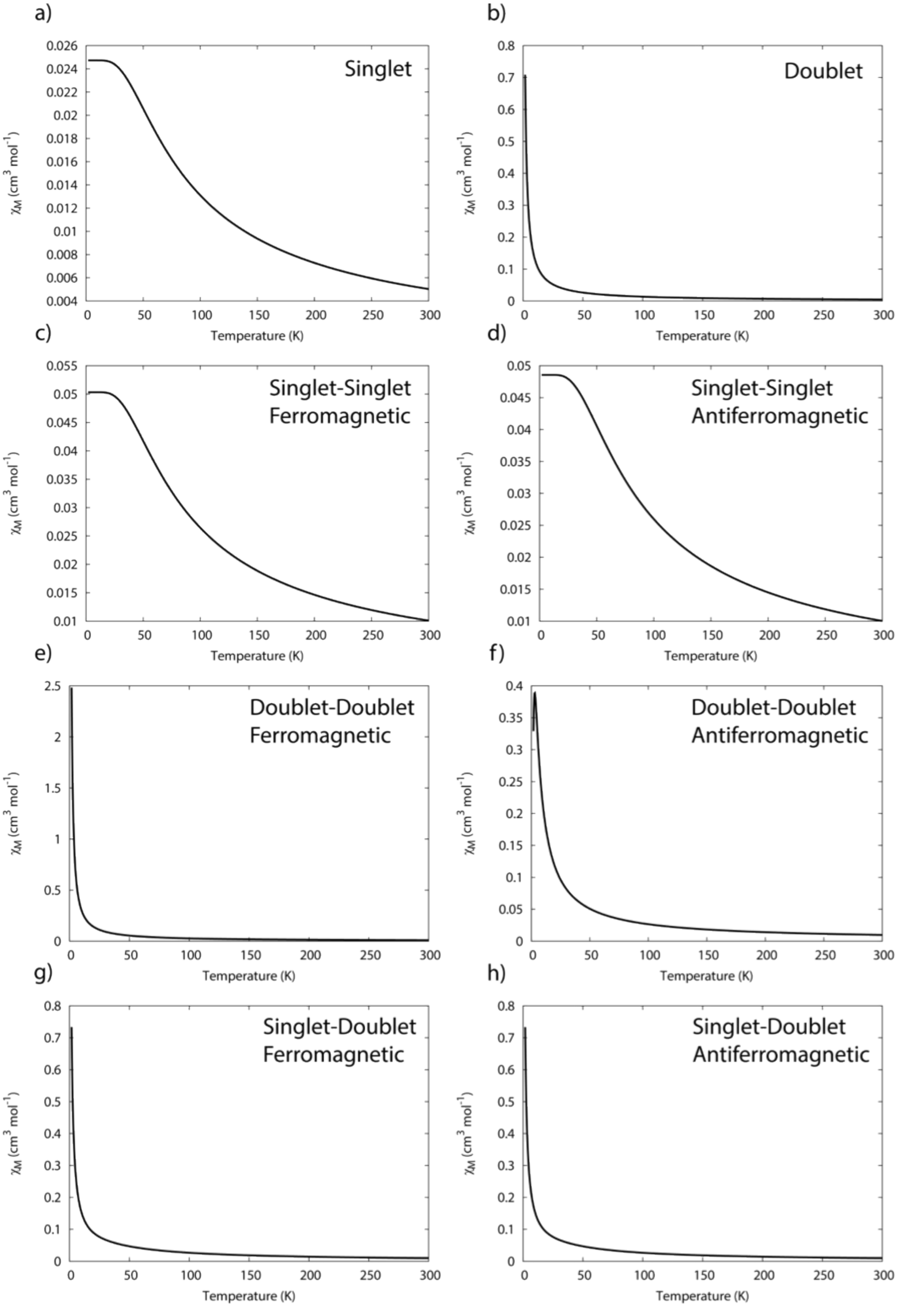

Figure S3. Temperature dependence of the magnetic susceptibility for model uranium(IV) complexes where $J = \pm 1$ cm^{-1}.

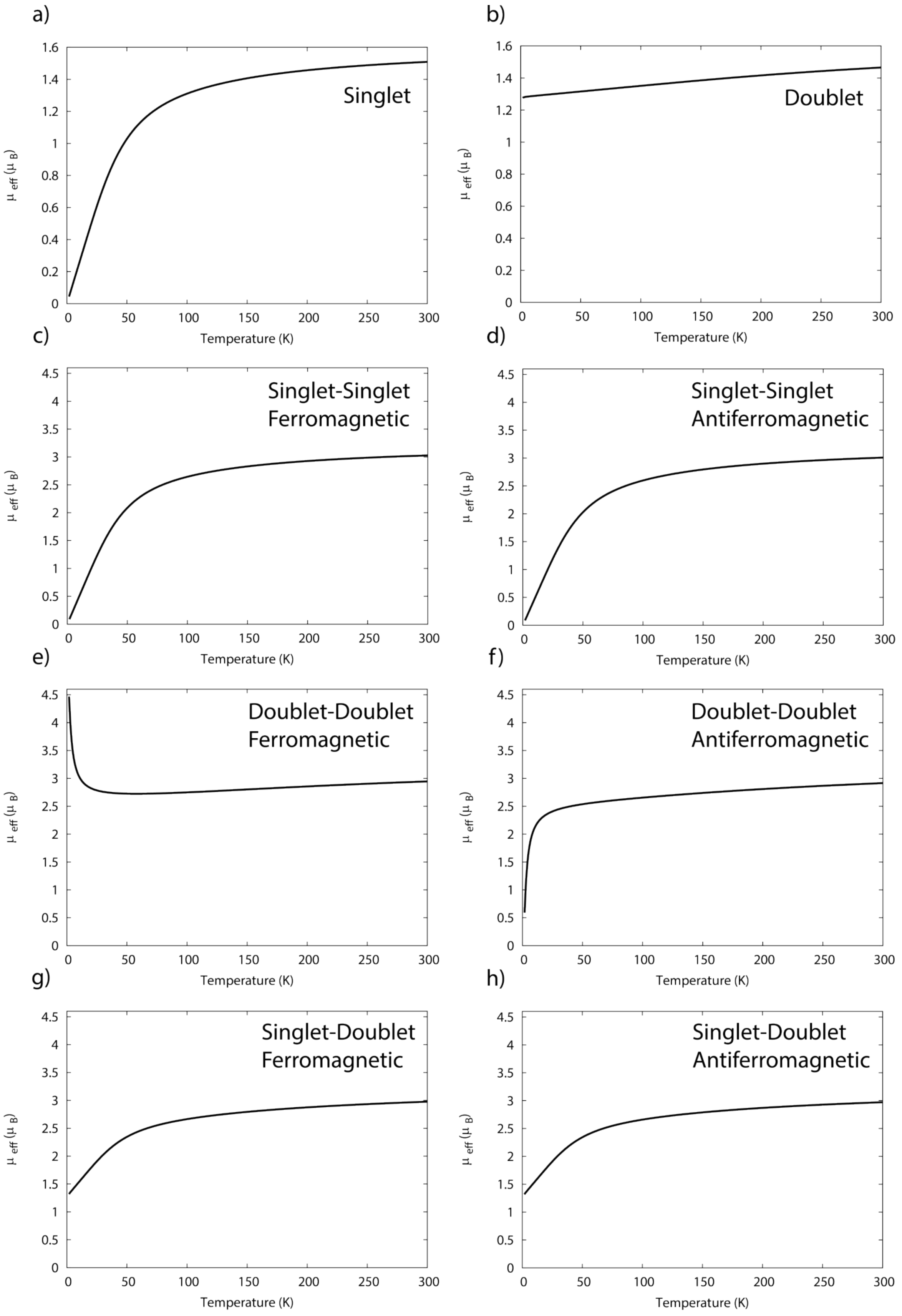

Figure S4. Temperature dependence of the magnetic moment for model uranium(IV) complexes where $J = \pm 1$ cm^{-1}.

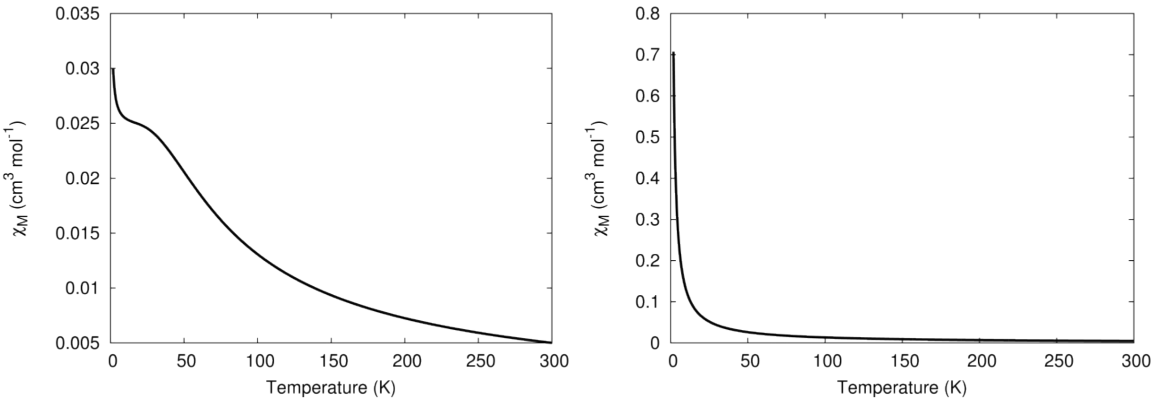

Figure S5. Temperature dependence of the magnetic susceptibility for monometallic model uranium(IV) complexes with a singlet (left) and pseudo-doublet (right) ground state, both with 1% $S = 1$ impurity.

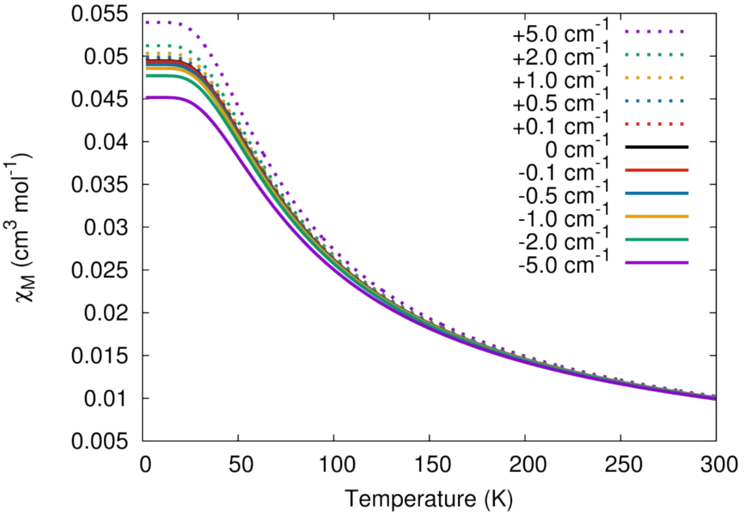

Figure S6. Temperature dependence of the magnetic susceptibility for dimetallic model uranium(IV) complexes, both sites having singlet ground states, as a function of the magnetic interaction J.

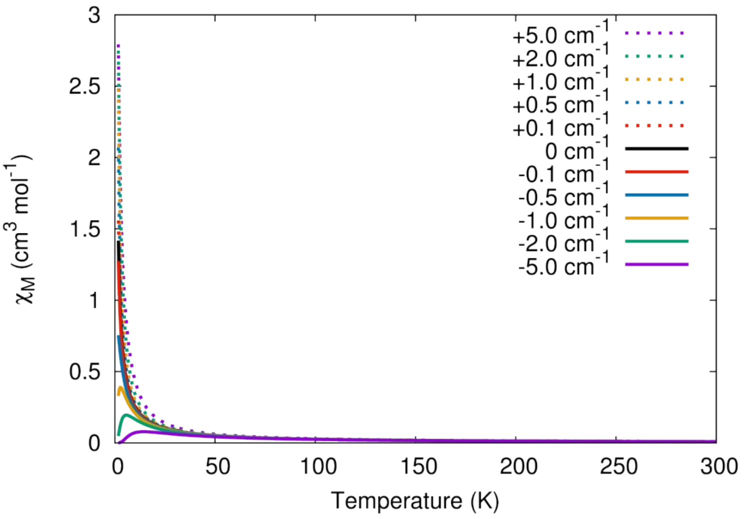

Figure S7. Temperature dependence of the magnetic susceptibility for dimetallic model uranium(IV) complexes, both sites having pseudo-doublet ground states, as a function of the magnetic interaction *J*.

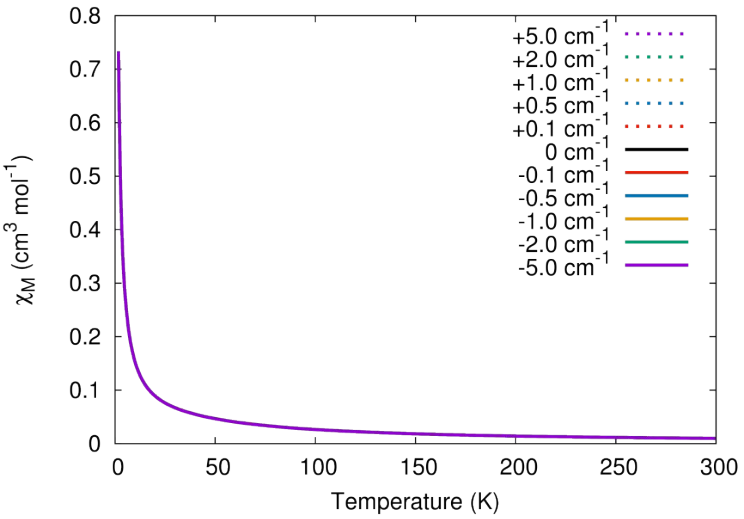

Figure S8. Temperature dependence of the magnetic susceptibility for dimetallic model uranium(IV) complexes, one site having a singlet ground state and the other a pseudo-doublet ground state, as a function of the magnetic interaction *J*.

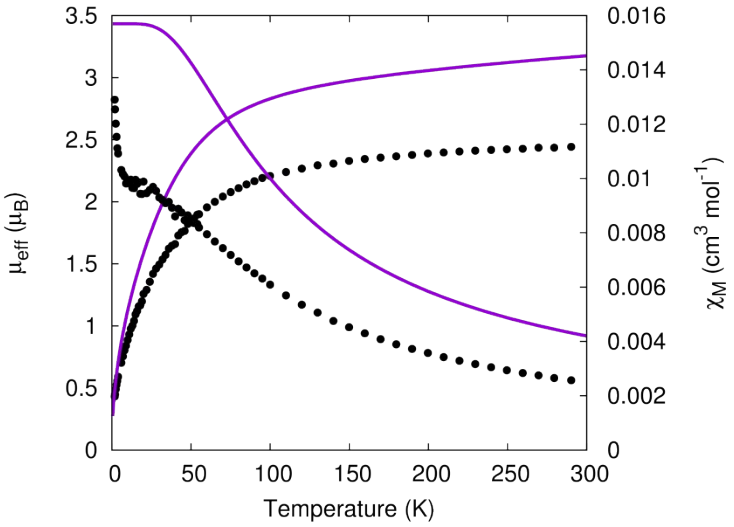

Figure S9. Temperature dependence of the magnetic moment for **2** (per uranium) measured in a 0.1 T field. Solid purple lines are simulations with Hamiltonian equation 3 with CF parameters from CASSCF-SO (Table S2) and $g_J = 0.80$.

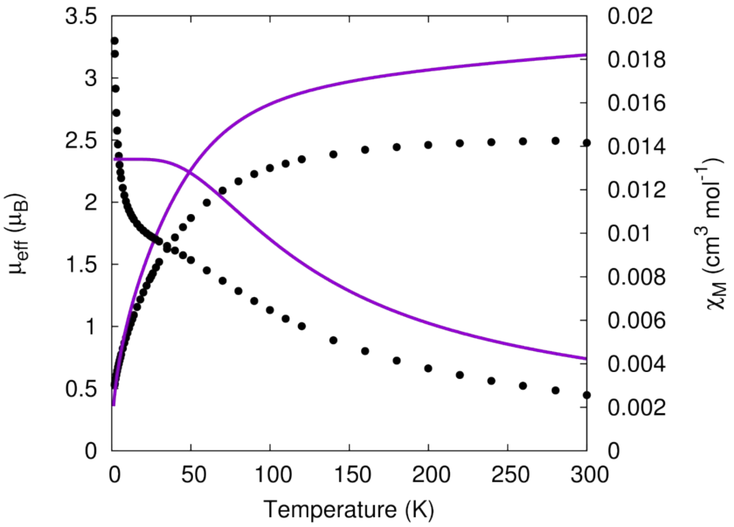

Figure S10. Temperature dependence of the magnetic moment for **5** (per uranium) measured in a 0.1 T field. Solid purple lines are simulations with Hamiltonian equation 3 with CF parameters from CASSCF-SO (Table S2) and $g_J = 0.80$.

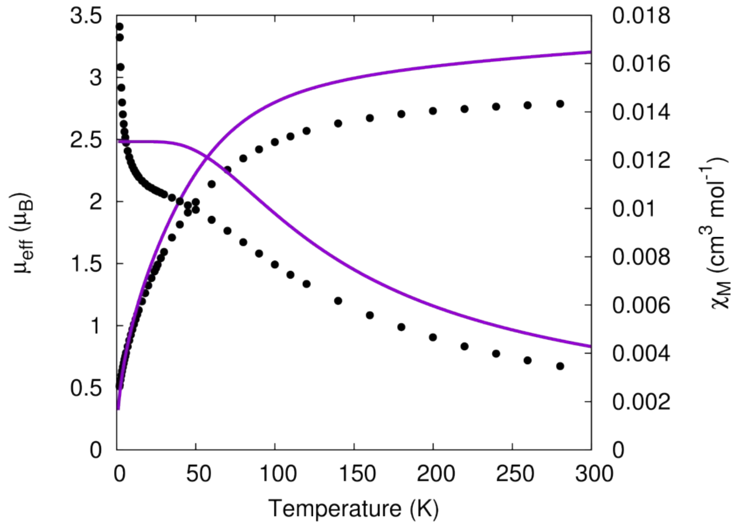

Figure S11. Temperature dependence of the magnetic moment for **6** (per uranium) measured in a 0.1 T field. Solid purple lines are simulations with Hamiltonian equation 3 with CF parameters from CASSCF-SO (Table S2) and $g_J = 0.80$.

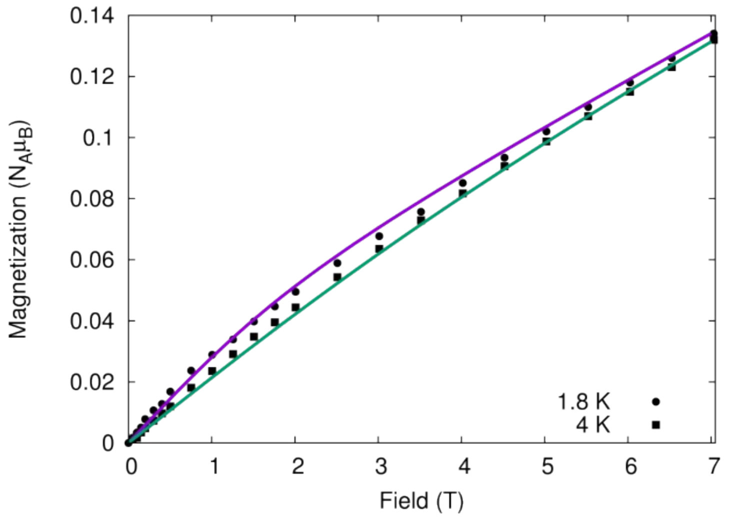

Figure S12. Field dependence of the magnetisation for **5** (per uranium). Solid lines are fits with Hamiltonian equation 3 with CF parameters from CASSCF-SO (Table S2) and those in Table 1.

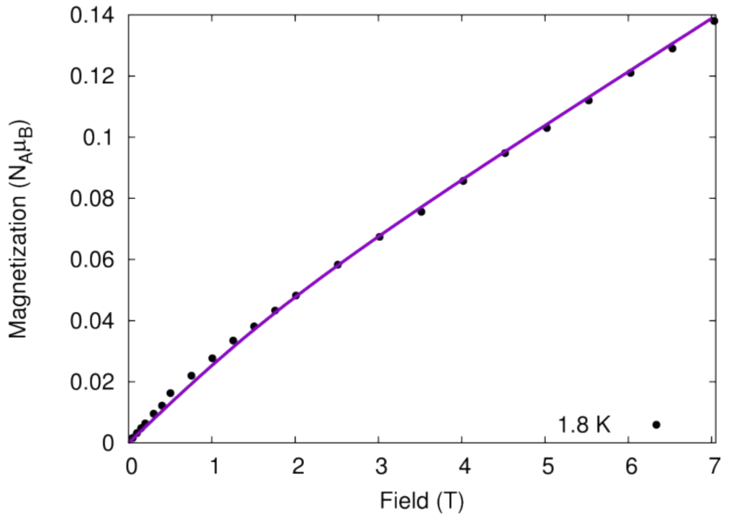

Figure S13. Field dependence of the magnetisation for **6** (per uranium). Solid line is a fit with Hamiltonian equation 3 with CF parameters from CASSCF-SO (Table S2) and those in Table 1.

Table S1. CASSCF-SO calculated CF splitting of the $J = 4$ SO multiplet for **2**. Subsequent excited states at ca. 6000 cm^{-1}, wavefunctions given to nearest per cent.

Energy (cm^{-1})	Wavefunction composition								
	$\lvert -4\rangle$	$\lvert -3\rangle$	$\lvert -2\rangle$	$\lvert -1\rangle$	$\lvert 0\rangle$	$\lvert +1\rangle$	$\lvert +2\rangle$	$\lvert +3\rangle$	$\lvert +4\rangle$
0	1	5	0	7	74	7	0	5	1
104	16	1	3	26	8	26	3	1	16
149	19	1	1	27	4	27	1	1	19
864	18	0	20	11	2	11	20	0	18
906	18	5	20	7	0	7	20	5	18
1162	8	3	24	15	1	15	24	3	8
1180	10	9	28	3	1	3	28	9	10
1300	9	36	3	3	0	3	3	36	9
1524	2	41	1	1	10	1	1	41	2

Table S2. CASSCF-SO calculated CF splitting of the $J = 4$ SO multiplet for **5**. Subsequent excited states at ca. 6000 cm^{-1}, wavefunctions given to nearest per cent.

Energy (cm^{-1})	Wavefunction composition								
	$\vert -4\rangle$	$\vert -3\rangle$	$\vert -2\rangle$	$\vert -1\rangle$	$\vert 0\rangle$	$\vert +1\rangle$	$\vert +2\rangle$	$\vert +3\rangle$	$\vert +4\rangle$
0	1	5	0	2	85	2	0	5	1
133	14	0	1	33	3	33	1	0	14
184	16	0	0	32	2	32	0	0	16
891	13	0	30	7	0	7	30	0	13
946	21	2	23	4	0	4	23	2	21
1193	17	1	23	9	0	9	23	1	17
1216	18	0	20	12	0	12	20	0	18
1302	1	48	1	0	0	0	1	48	1
1540	0	44	1	0	10	0	1	44	0

Table S3. CASSCF-SO calculated CF splitting of the $J = 4$ SO multiplet for **6**. Subsequent excited states at ca. 6000 cm^{-1}, wavefunctions given to nearest per cent.

Energy (cm^{-1})	Wavefunction composition								
	$\vert -4\rangle$	$\vert -3\rangle$	$\vert -2\rangle$	$\vert -1\rangle$	$\vert 0\rangle$	$\vert +1\rangle$	$\vert +2\rangle$	$\vert +3\rangle$	$\vert +4\rangle$
0	1	3	1	17	55	17	1	3	1
152	9	2	7	18	29	18	7	2	9
195	11	1	6	32	2	32	6	1	11
907	6	5	28	11	1	11	28	5	6
961	4	7	33	4	1	4	33	7	4
1314	11	25	13	1	2	1	13	25	11
1324	24	8	3	13	4	13	3	8	24
1417	29	13	4	3	1	3	4	13	29
1645	5	35	5	2	7	2	5	35	5

Table S4 CASSCF-SO calculated g_J-values, LoProp charges on E atoms and percentage of the active space made up from E-based AOs for complexes **2**, **5** and **6**.

**Parameter**	**2**	**5**	**6**
g_J	0.764	0.765	0.763
LoProp charge on E	-1.40	-1.41	-1.42
%E AOs in active space	2.42	2.39	1.35

Table S5. CASSCF-SO calculated CF parameters for the J = 4 SO multiplet for complexes **2**, **5** and **6**.

Parameter	**2** (cm^{-1})	**5** (cm^{-1})	**6** (cm^{-1})
B_2^{-2}	2.3289148041640E+00	-2.9914085051278E+00	5.7757063011451E-01
B_2^{-1}	3.3426231592011E+00	-1.7036877849463E+00	1.2948252582294E+01
B_2^0	7.6900705046281E+00	9.8890014880665E+00	1.5122309588220E+01
B_2^1	-9.4293156257446E-01	5.7758482415478E+00	1.0451220838012E+01
B_2^2	-1.9073705297228E+00	-2.5649393927059E+00	-1.6400282256839E+00
B_4^{-4}	2.3577805036148E-01	5.9405415479970E-02	4.7349234038220E-02
B_4^{-3}	-3.4360771402728E+00	-2.8408768132648E+00	6.2793605421221E+00
B_4^{-2}	-1.6813101312674E-01	9.0472554550677E-02	-6.8314164447387E-01
B_4^{-1}	4.4135223613976E-01	5.0359696993235E-02	-1.3045801518726E+00
B_4^0	-3.6373099128587E-01	-3.8086156201822E-01	-2.7667653356584E-01
B_4^1	7.4161422467791E-01	-3.6439373044571E-01	-2.1229397168961E-01
B_4^2	-4.1224700348751E-01	-1.8025812637644E-01	-2.9819737585125E-01
B_4^3	-5.9152394828630E+00	6.1435644145974E+00	-2.1620241536673E+00
B_4^4	9.7956245490351E-02	-3.8112733169346E-03	-5.2584663336904E-01
B_6^{-6}	-2.4153390211119E-02	2.2328090187307E-02	2.0928365451043E-02
B_6^{-5}	-1.6278199789860E-02	-8.6268571523176E-03	7.2259902455398E-03
B_6^{-4}	-1.2571249909051E-03	-6.2601703261622E-04	5.0841951783438E-04
B_6^{-3}	-1.4544735834047E-02	-9.9639778207340E-03	2.1596645238169E-02
B_6^{-2}	-7.1225355164836E-04	1.9474926446661E-03	-1.1277560979534E-02
B_6^{-1}	-4.6886205324651E-03	-1.7197060458220E-03	-8.0724109543613E-05
B_6^0	1.4811080814430E-03	1.5328830449610E-03	3.6237633566770E-04
B_6^1	-7.8660143440975E-03	1.3819209587061E-03	9.4162265818005E-03
B_6^2	-6.2025803292708E-03	-3.7558791982058E-03	-8.2091515644682E-03
B_6^3	-2.5086977135773E-02	3.1645088440075E-02	-2.2880430357666E-02
B_6^4	1.6199523688113E-03	2.3381021954171E-03	-5.1877520935448E-03
B_6^5	-1.2531366643478E-02	-6.3907827062485E-04	-3.1252987090844E-02
B_6^6	-1.8567781142606E-02	-2.0377573943464E-02	3.0735474279598E-02
